# The blood proteome predicts the impact of circulating factors on age-related mitochondrial health

**DOI:** 10.1038/s41514-026-00449-8

**Published:** 2026-07-24

**Authors:** Stephanie R. Heimler, Jaclyn Bergstrom, Nina S. Sun, Toshiko Tanaka, Ann Zenobia Moore, Julián Candia, Brian H. Chen, Qu Tian, Arsun Bektas, Giovanna Fantoni, Christopher Dunn, Mary Kaileh, Ranjan Sen, Luigi Ferrucci, Anthony J. A. Molina

**Affiliations:** 1 University of California San Diego, School of Medicine, La Jolla, CA USA; 2 Translational Gerontology Branch, National Institute on Aging, Baltimore, MD USA; 3 California Pacific Medical Center Research Institute, San Francisco, CA USA; 4 Clinical Research Core, National Institute on Aging, Baltimore, MD USA; 5 Flow Cytometry Core, National Institute on Aging, Baltimore, MD USA; 6 Laboratory of Molecular Biology and Immunology, National Institute on Aging, Baltimore, MD USA; 7 Sam and Rose Stein Institute for Research on Aging, University of California, La Jolla, CA USA

**Keywords:** Biomarkers, Cell biology, Computational biology and bioinformatics

## Abstract

Circulating non-cellular factors, such as plasma proteins, contribute to various features of aging. To determine the impacts of endogenous circulating factors on human age-related bioenergetic decline, we treated primary human fibroblasts with serum samples representing the adult life-course. Our results demonstrate that the maximal mitochondrial bioenergetic capacity of fibroblasts treated with serum is negatively correlated with the chronological and epigenetic age of the serum donor. Using targeted proteomics, we identified plasma proteins associated with the bioenergetic effects of serum. We then utilized elastic net, a linear regression modeling technique, to derive a novel proteomic signature of age-related mitochondrial differences. MitoAge is a 25-protein signature of age-related mitochondrial health that predicts the systemic bioenergetic effects of circulating factors and is related to differences in physical function across human aging. Signatures that report on cellular hallmarks of aging, such as mitochondrial function, represent a new generation of mechanistically-informed biomarkers of biological aging.

**Figure:**
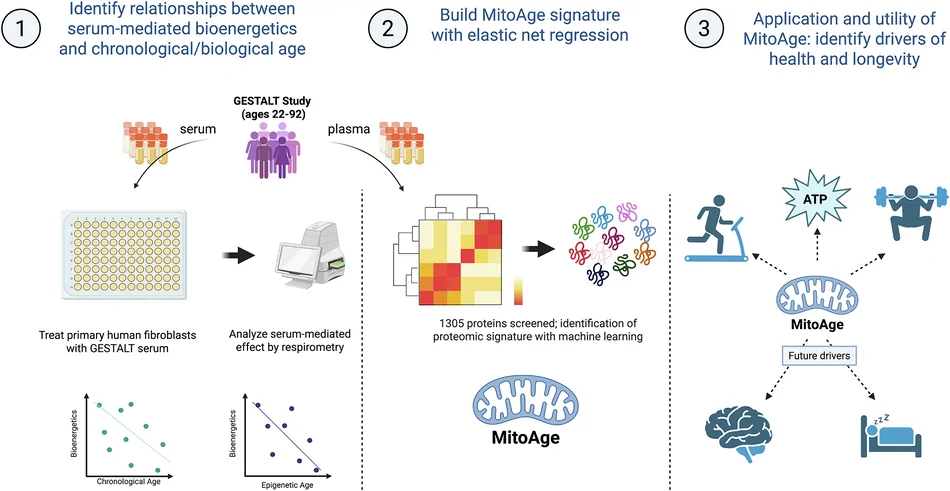

## Introduction

Aging is one of the strongest predictors of chronic disease, frailty, and a number of adverse conditions that negatively impact older adults. Mitochondrial dysfunction is a widely recognized cellular hallmark of aging^1^, and can drive age-related cellular processes^2–5^, functional decline^6^, and cognitive impairment^7^. We and others have shown that age-related mitochondrial differences are systemic, as exhibited by the respirometric profiles of circulating blood cells^8–10^.

Multiple lines of evidence demonstrate that blood harbors factors that can drive aging across organ systems, including skeletal muscle^11^, liver^12^, heart^13^, and pancreas^14^. Heterochronic parabiosis studies in mice have demonstrated that circulating factors in blood drive multiple features of aging, including mitochondrial dysfunction. Using this model, our group previously reported that blood can mediate skeletal muscle age-related bioenergetic decline^15^. Across measures of mitochondrial function, young mice in parabiosis with older mice exhibited mitochondrial deficits associated with aging. Importantly, we observed an equalization of bioenergetic capacities between young and old heterochronic pairs, clearly demonstrating that circulating factors are the primary drivers of age-related bioenergetic differences. We have also reported that circulating factors play a central role in mitochondria changes associated with behavioral interventions, such as diet and exercise^16^ and age-related disease, such as Alzheimer’s disease and dementia^17,18^. Based on these previous studies, we postulated that in-depth analysis of circulating factors could enable the prediction of systemic age-related bioenergetic decline. Therefore, using plasma from individuals representing the adult human life-course, we sought to identify circulating protein signatures that are predictive of age-associated differences in mitochondrial function.

In recent years, factors found in circulation, such as lipids, metabolites, and proteins, have been associated with chronological age^19–23^. Among the types of molecular analytes that can serve as biomarkers, proteins have been shown to be particularly useful, and previous studies have already shown that circulating proteins can serve as promising biomarkers of age and disease^24–28^. In order to identify circulating factors that are directly associated with the effects on mitochondrial function, we first treated naïve human primary fibroblast cells with serum from participants representing the adult human lifespan to investigate how mitochondrial oxidative phosphorylation (OXPHOS) correlated with both chronological and epigenetic age of the participants from whom the serum samples were derived. We then conducted proteomic analysis of the serum samples to identify circulating proteins that were most correlated with the bioenergetic effects of these samples. To test the hypothesis that circulating proteins drive the effects of serum on cellular bioenergetics, we utilized computational approaches to develop a signature based on proteomic data alone, and examined how accurately we could predict the bioenergetic effects of whole serum (**Graphical Abstract**). The age-related proteomic signature of mitochondrial health (MitoAge) was trained to predict the bioenergetic effects of serum derived from individuals 22 to 92 years of age. Given the recognized role of mitochondria in aging and a wide array of age-related conditions, including physical function decline^29–31^, we then examined associations between MitoAge with strength and fitness. The development of signatures that report on cellular hallmarks of aging advances efforts to develop mechanistically informed biomarkers of biological aging. MitoAge was developed to report on age-related bioenergetic differences, relying on the effect of age as part of the model. The goal of MitoAge is to be used across multiple studies to inform on the role of age-related bioenergetic differences and broaden our understanding of drivers of health and longevity.

## Results

### Participant demographics

The ages of the 88 participants in this analytical group were between 22 and 92 (mean: 54.14 years), with body mass index (BMI) ranging from 19.6 to 31.0 (mean 25.8), and 42% were female.

### Serum-mediated mitochondrial bioenergetics negatively correlate with the chronological ages of the serum donors

**Fig. 1:**
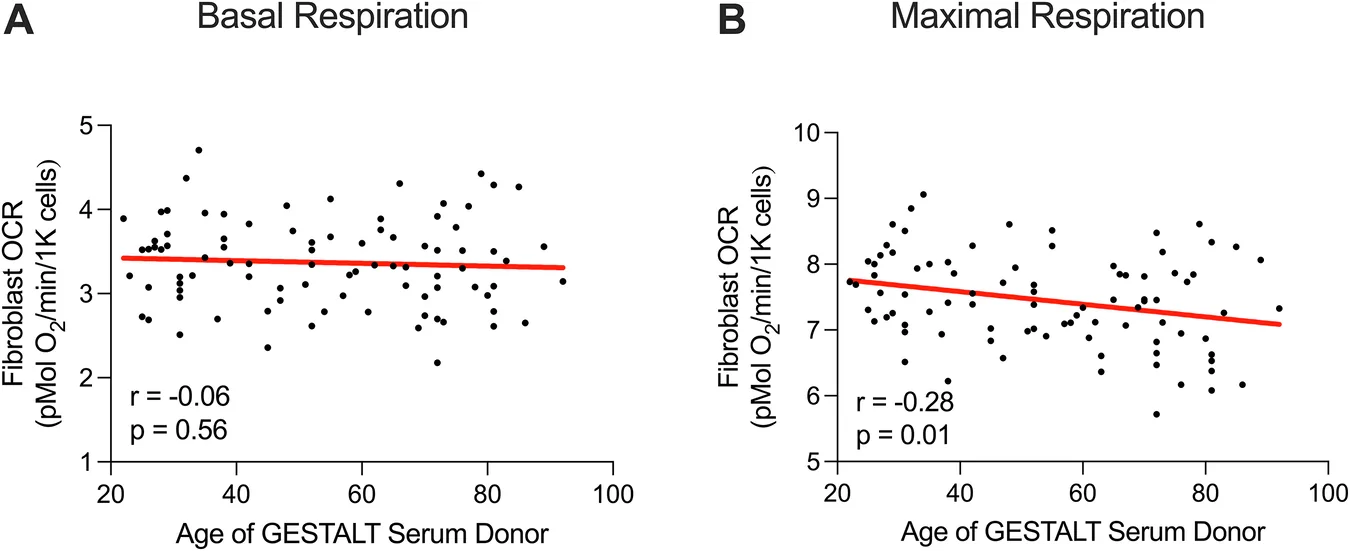
Maximal respiration, but not basal respiration, negatively correlates with the chronological age of the serum donor. *N* = 88 GESTALT serum samples treated on human primary fibroblasts (fibroblast donor = male, 33 y.o). Basal respiration (**A**) or maximal respiration (**B**) of the serum-mediated bioenergetic effect are shown.

We measured the bioenergetic capacity of primary human fibroblasts (33 y.o. donor) treated with GESTALT serum samples to assess how extrinsic factors in serum may cause differences in bioenergetics of naïve cells. We observed no significant relationship between the chronological age of the GESTALT serum donor and fibroblast basal respiration after serum treatment (R = 0.06, *P* = 0.56; Fig. 1A). We found a significant correlation between the age of the GESTALT serum donors and maximal respiration after serum treatment (R = –0.28, *P* = 0.01; Fig. 1B).

### Serum-mediated bioenergetic effects negatively relate to epigenetic age of serum donors in multiple tissue types

**Fig. 2:**
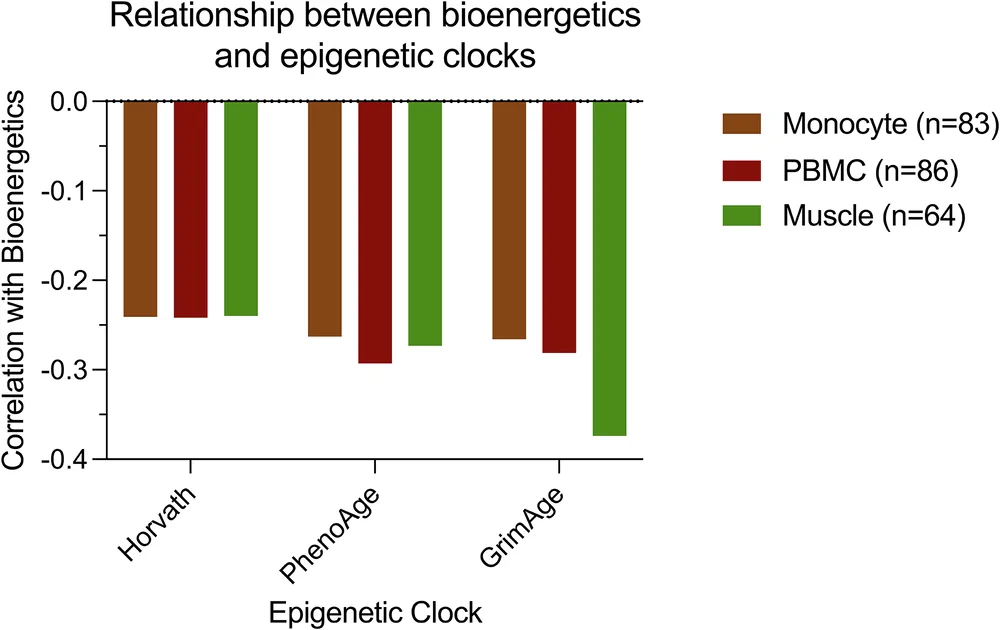
Serum-mediated bioenergetic effects relate to the epigenetic age of serum donors in multiple tissue types. Serum-mediated bioenergetic effects are based on the maximal respiration of young fibroblast donor.

To investigate if the bioenergetic effects of serum were more strongly associated with biological age than chronological age, we compared the correlations between fibroblast serum-mediated bioenergetics and epigenetic ages derived from PBMCs, monocytes, and muscle tissue from GESTALT participants (Fig. 2). Epigenetic ages were calculated for the Horvath (pan-tissue), DNAmPhenoAge, and GrimAge clocks, as these represent potentially different aspects of aging (molecular, phenotypic, or mortality-focused). We observed that the serum-mediated bioenergetic metric and the Horvath clock were negatively correlated at the same magnitude across all three cell types (*R* = –0.24). Second-generation epigenetic clocks, DNAmPhenoAge and GrimAge, were more strongly negatively correlated to serum-mediated bioenergetics compared to the Horvath clock for all cell or tissue types. We observed similar correlations between serum-mediated bioenergetics and DNAmPhenoAge of monocytes, PBMCs, and muscle tissue (R = –0.26; *R* = –0.29; R = –0.27, respectively). With GrimAge, differences in the correlations with monocytes, PBMCs, and muscle tissue (R = –0.27; R = –0.28; R = –0.37, respectively) were notable. Overall, the correlations between bioenergetics and the Horvath clock and the PhenoAge clock were very consistent regardless of tissue type (i.e., between monocytes, PBMCs, and muscle tissue), whereas the correlations involving the GrimAge clock have more variation depending on the tissue from which methylation data were derived. Upon adjusting these relationships for participant chronological age, we observed that these associations were attenuated (Supplementary Table 1), as would be expected as these epigenetic clocks are strongly correlated with chronological age^32–34^.

### Identification of circulating proteins related to serum-mediated bioenergetics

**Fig. 3:**
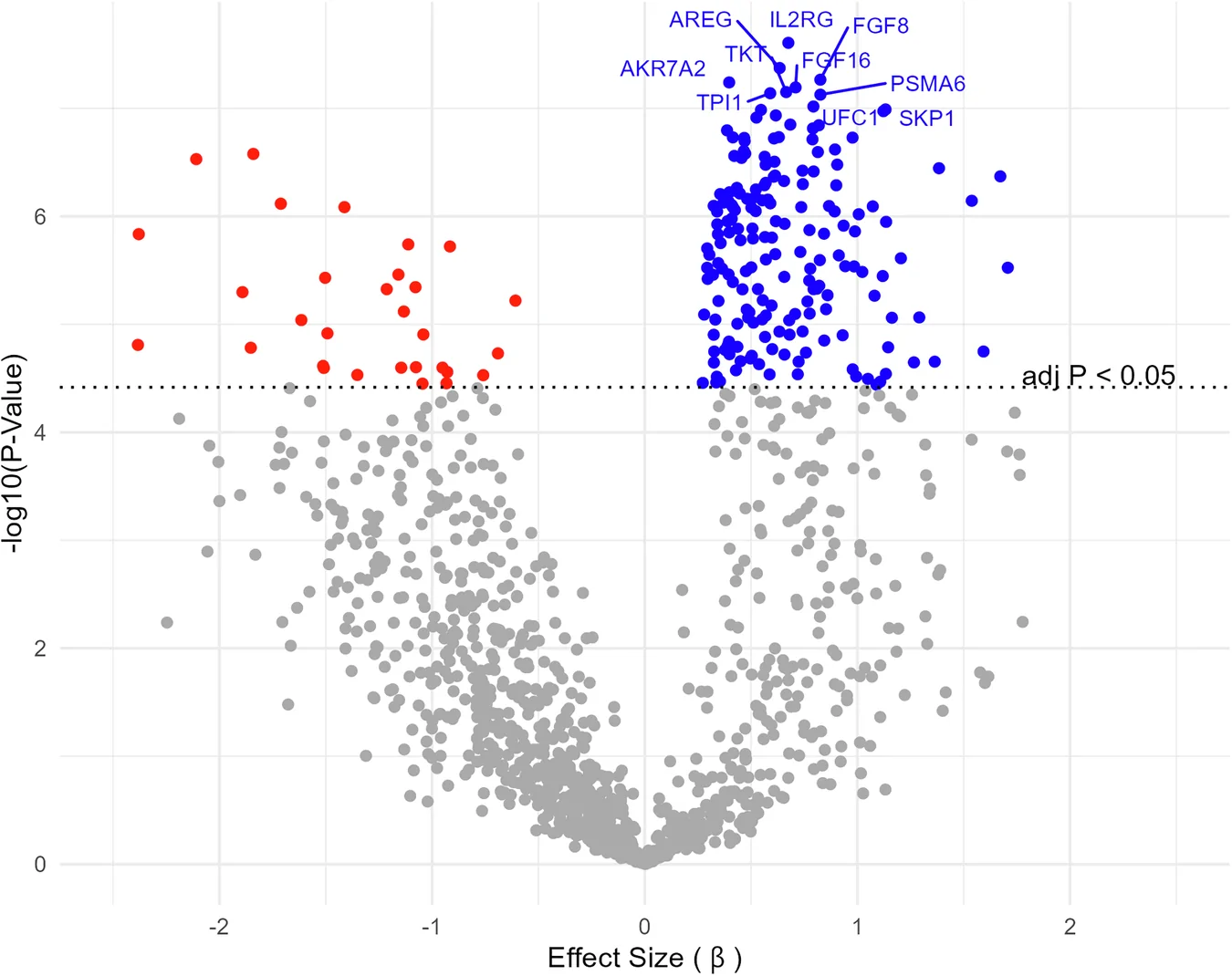
Serum proteins are positively (*n* = 183; blue) and negatively (*n* = 32; red) associated with the bioenergetic effects of serum using univariate linear regression models. Results are adjusted for sex and batch; used Bonferroni correction.

We used univariate linear regression models to evaluate the abundance of circulating proteins in plasma correlated with the serum-mediated bioenergetics. Of the 1305 proteins tested, 215 proteins were significantly associated with serum-mediated bioenergetics (Bonferroni corrected *P* < 3.83 × 10^–5^). There were 183 positively associated proteins and 32 negatively associated proteins, as depicted by the volcano plot in Fig. 3. The top 25 proteins ranked by *P* value are presented in Table 1, with an expanded table of all 1305 proteins available in Supplementary Data 1. The protein with the strongest association with serum-mediated bioenergetics was interleukin 2 receptor subunit gamma (IL-2 sRg, Beta = 0.6751, *P* < 0.001). In addition to IL-2 sRg, the top 10 most significant proteins were amphiregulin (AREG), fibroblast growth factor 8 A (FGF-8A), Aflatoxin B1 aldehyde reductase (AKR7A2), fibroblast growth factor 16 (FGF-16), transketolase (TKT), triosephosphate isomerase (TPI1), proteasome subunit alpha type-6 (PSA6), ubiquitin-fold modifier conjugating enzyme 1 (UFC1), and S-phase kinase-associated protein 1 (SKP1).

**Table 1:** Top 25 most significant proteins associated with maximal respiration of the young fibroblast donor treated with GESTALT serum

Seq ID	Gene ID	UniProt ID	Target	Beta	SE	*P* value
**2634-2**	**IL2RG**	**P31785**	**IL-2 sRg**	**0.6751**	**0.1093**	**2.47E–08**
**2970-60**	**AREG**	**P15514**	**AREG**	**0.6344**	**0.1048**	**4.23E–08**
**4394-71**	**FGF8**	**P55075**	**FGF-8A**	**0.8257**	**0.1378**	**5.43E–08**
4188-1	AKR7A2	O43488	Aflatoxin B1 aldehyde reductase	0.3970	0.0664	5.76E–08
**4393-3**	**FGF16**	**O43320**	**FGF-16**	**0.7094**	**0.1191**	**6.40E–08**
4306-4	TKT	P29401	Transketolase	0.6652	0.1121	7.07E–08
4309-59	TPI1	P60174	Triosephosphate isomerase	0.5899	0.0995	7.25E–08
**3860-7**	**PSMA6**	**P60900**	**PSA6**	**0.8260**	**0.1395**	**7.46E–08**
**3405-6**	**UFC1**	**Q9Y3C8**	**UFC1**	**0.7931**	**0.1354**	**9.61E–08**
3902-21	SKP1	P63208	SKP1	1.1324	0.1938	1.02E–07
**4963-19**	**ARPP19**	**P56211**	**ARP19**	**0.5466**	**0.0936**	**1.03E–07**
4547-59	FLRT1	Q9NZU1	FLRT1	1.1223	0.1923	1.06E–07
3897-61	PDXP	Q96GD0	PLPP	0.6162	0.1060	1.16E–07
**4249-64**	**NME2**	**P22392**	**NDP kinase B**	**0.5251**	**0.0905**	**1.22E–07**
**3874-8**	**UBE2L3**	**P68036**	**UB2L3**	**0.6844**	**0.1187**	**1.41E–07**
5888-29	EIF5A	P63241	eIF-5A-1	0.8188	0.1420	1.43E–07
**4389-2**	**DHH**	**O43323**	**DHH**	**0.7911**	**0.1376**	**1.53E–07**
5346-24	CPNE1	Q99829	CPNE1	0.3868	0.0674	1.60E–07
**10363-13**	**SMAD3**	**P84022**	**SMAD3**	**0.6303**	**0.1105**	**1.85E–07**
3593-72	CASP3	P42574	Caspase-3	0.4143	0.0727	1.86E–07
4396-54	IFNL1	Q8IU54	IFN-lambda 1	0.9770	0.1714	1.87E–07
3817-18	PRKCQ	Q04759	KPCT	0.4678	0.0821	1.88E–07
4258-15	PA2G4	Q9UQ80	PA2G4	0.6078	0.1067	1.90E–07
3877-67	CAMKK1	Q8N5S9	CaMKK alpha	0.7888	0.1386	1.94E–07
3848-14	GAPDH	P04406	GAPDH, liver	0.4699	0.0827	2.01E–07

Protein targets (SOMAmers) are ranked by beta coefficients in this univariate analysis. Proteins in bold type indicate that they overlap with protein targets identified in the multivariate analysis.

### Age-related proteomic signature predicts the bioenergetic effects of circulating factors

**Fig. 4:**
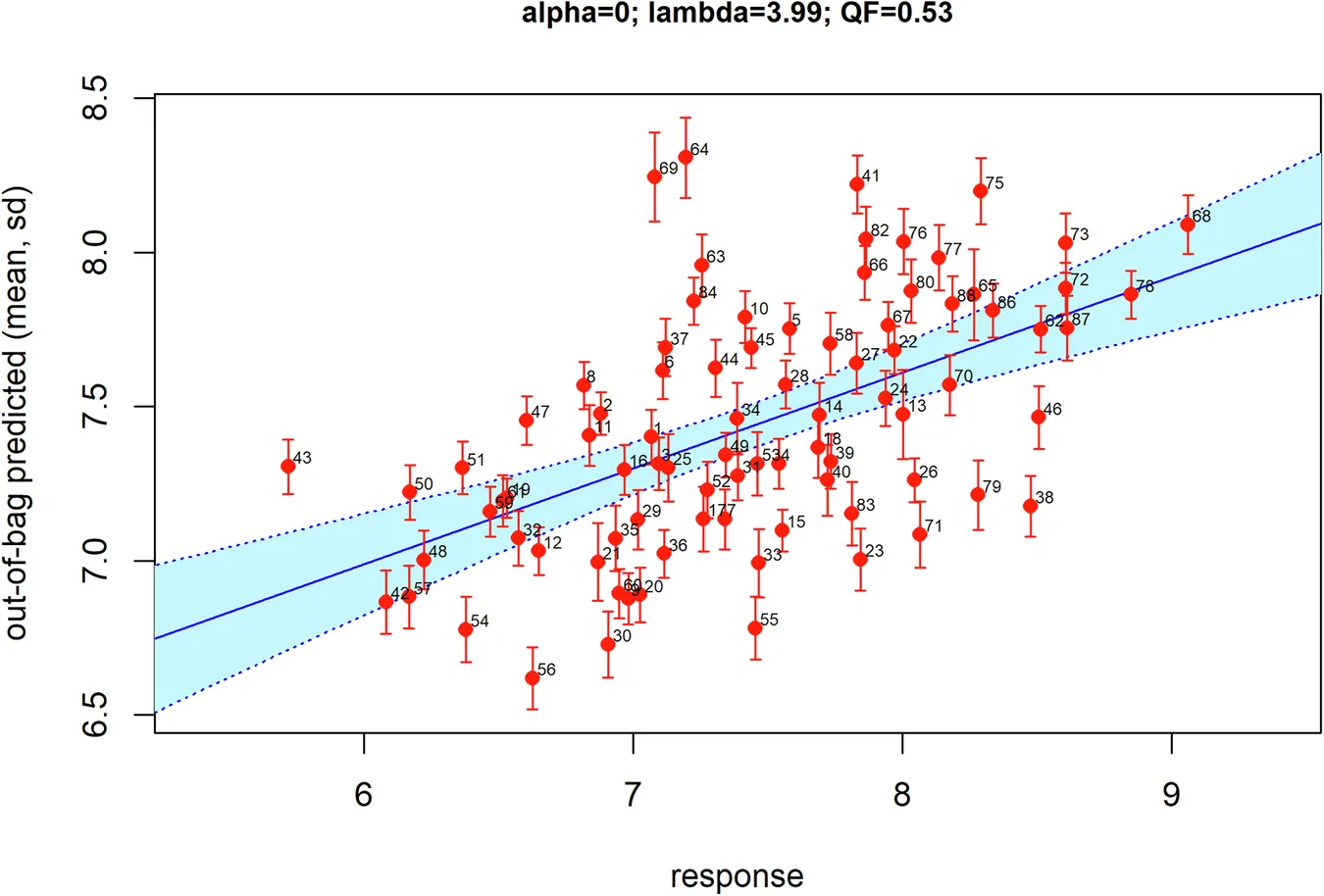
Precision and accuracy of MitoAge. Correlation between the actual (observed) maximal respiration from serum treatment versus the predicted maximal respiration using the proteomic signature. An elastic net model was assessed using eNetXplorer^67^ with parameters n_run = 500, n_perm_null = 125, n_fold = 5. Using Pearson’s correlation between the actual maximal respiration (x-axis) and out-of-bag predicted rmaximal respiration (y-axis) as quality factor (QF), the optimal model was defined by mixing parameter alpha = 0 and shrinkage parameter lambda = 3.99. Symbols represent individual participants; error bars represent the range of variation (standard deviation) of predicted values.

To derive MitoAge, we fit an elastic net regression model to identify a set of protein markers associated with the bioenergetic effect of serum on young fibroblasts. The elastic net model accuracy is depicted in Fig. 4, which shows the correlation between the out-of-bag prediction and actual maximal respiration for each participant. The selected elastic net mixing parameter alpha(0) and penalty parameter lambda (3.99) are also shown. The intercept is 7.497. The elastic net identified 25 proteins associated with maximal respiration (Table 2). The protein with the strongest association with serum-mediated bioenergetics was C-C motif chemokine 2 (MCP-1, Weight = –0.433, *P* = 0.021). In addition to MCP-1, the top 10 most significant proteins identified from the elastic net included AREG, cathepsin D (CTSD), interferon alpha-2 (IFN-aA), IL-2 sRg, mothers against decapentaplegic homolog 3 (SMAD3), tartrate-resistant acid phosphatase type 5 (TrATPase), tumor necrosis factor ligand superfamily member 13B (BAFF), FGF-16, and Dickkopf-related protein 1 (DKK1). Mediation analysis estimated that 91% of the age-associated difference in serum-mediated bioenergetics was statistically accounted for by MitoAge (Table 3), suggesting that the mitochondrial aging signature captured by MitoAge is a primary mechanism through which chronological age influences cellular bioenergetics. Out of these proteins identified by the elastic net, 11 of them overlapped with the univariate analysis (Table 2, bolded type). The overlapping proteins are: IL-2 sRg, UFC1, FGF-16, cAMP-regulated phosphoprotein 19 (ARP19), AREG, SMAD3, ubiquitin conjugating enzyme E2 L3 (UB2L3), desert hedgehog protein (DHH), PSA6, FGF-8A, and nucleoside diphosphate kinase B (NME2).

**Table 2:** MitoAge features

Seq ID	Gene ID	UniProt ID	Target	Weight
2578-67	CCL2	P13500	MCP-1	–0.433
**2970-60**	**AREG**	**P15514**	**AREG**	**–0.002**
5508-62	CTSD	P07339	Cathepsin D	–0.45
3497-13	IFNA2	P01563	IFN-aA	–0.443
**2634-2**	**IL2RG**	**P31785**	**IL-2 sRg**	**0.025**
**10363-13**	**SMAD3**	**P84022**	**SMAD3**	**0.011**
3232-28	ACP5	P13686	TrATPase	–0.416
3059-50	TNFSF13B	Q9Y275	BAFF	–0.257
**4393-3**	**FGF16**	**O43320**	**FGF-16**	**–0.009**
3535-84	DKK1	O94907	DKK1	0.164
2630-12	IL1RAP	Q9NPH3	IL-1 R AcP	0.271
2826-53	EDA	Q92838	EDA	0.275
5460-60	DDX19B	Q9UMR2	DEAD-box protein 19B	0.052
**4249-64**	**NME2**	**P22392**	**NDP kinase B**	**0.024**
2944-66	NBL1	P41271	DAN	–0.294
10344-334	IL10RA	Q13651	IL-10 Ra	–1.053
**3860-7**	**PSMA6**	**P60900**	**PSA6**	**0.016**
3810-50	FGR	P09769	FGR	–0.14
**4389-2**	**DHH**	**O43323**	**DHH**	**0.036**
**3874-8**	**UBE2L3**	**P68036**	**UB2L3**	**0.084**
**3405-6**	**UFC1**	**Q9Y3C8**	**UFC1**	**0.107**
**4963-19**	**ARPP19**	**P56211**	**ARP19**	**0.006**
4549-78	FUT5	Q11128	FUT5	–0.055
**4394-71**	**FGF8**	**P55075**	**FGF-8A**	**0.118**
5245-40	PRKAA2 PRKAB2 PRKAG1	P54646 O43741 P54619	AMPK a2b2g1	0.01
			Intercept	7.497
			Sex (Male)	–0.12

Proteins (or sex) are listed in order of *P* value (corresponding with Supplementary Data 1) in this multivariate analysis. Proteins in bold type indicate that they overlap with protein targets identified in the univariate analysis from Table 1. The directionality of the model coefficients (the “Weight”) reflects the direction of association between each predictor and the outcome within the multivariable elastic net model. Collectively, the 25 proteins and sex were selected as part of the predictive model of the signature.

**Table 3:** Mediation analysis to examine relationships between age and bioenergetics

	Estimate	95% CI	*p* value
ACME (Average Causal Mediation Effect)	–0.009	–0.016, –0.002	0.006
ADE (Average Direct Effect)	–0.009	–0.005, 0.003	0.697
Total Effect	–0.010	–0.018, –0.002	0.012
Proportion Mediated	0.913	0.513, 1.656	0.010

Indirect, direct, and total effects are shown, and were estimated using nonparametric bootstrapping with 5000 simulations and percentile-based 95% confidence intervals.

### Serum mediates bioenergetic differences regardless of fibroblast donor age

In order to assess the potential influence of intrinsic cellular age on serum-mediated bioenergetics, we repeated the serum exposure on fibroblasts from an older donor (72 y.o. donor, Supplementary Fig. 1). Similar to the serum treatment of young fibroblast cells (33 y.o. donor), we found a significant correlation between the age of the GESTALT serum donors and maximal respiration after serum treatment (R = –0.25, *P* = 0.02).

### Testing of MitoAge signature

**Fig. 5:**
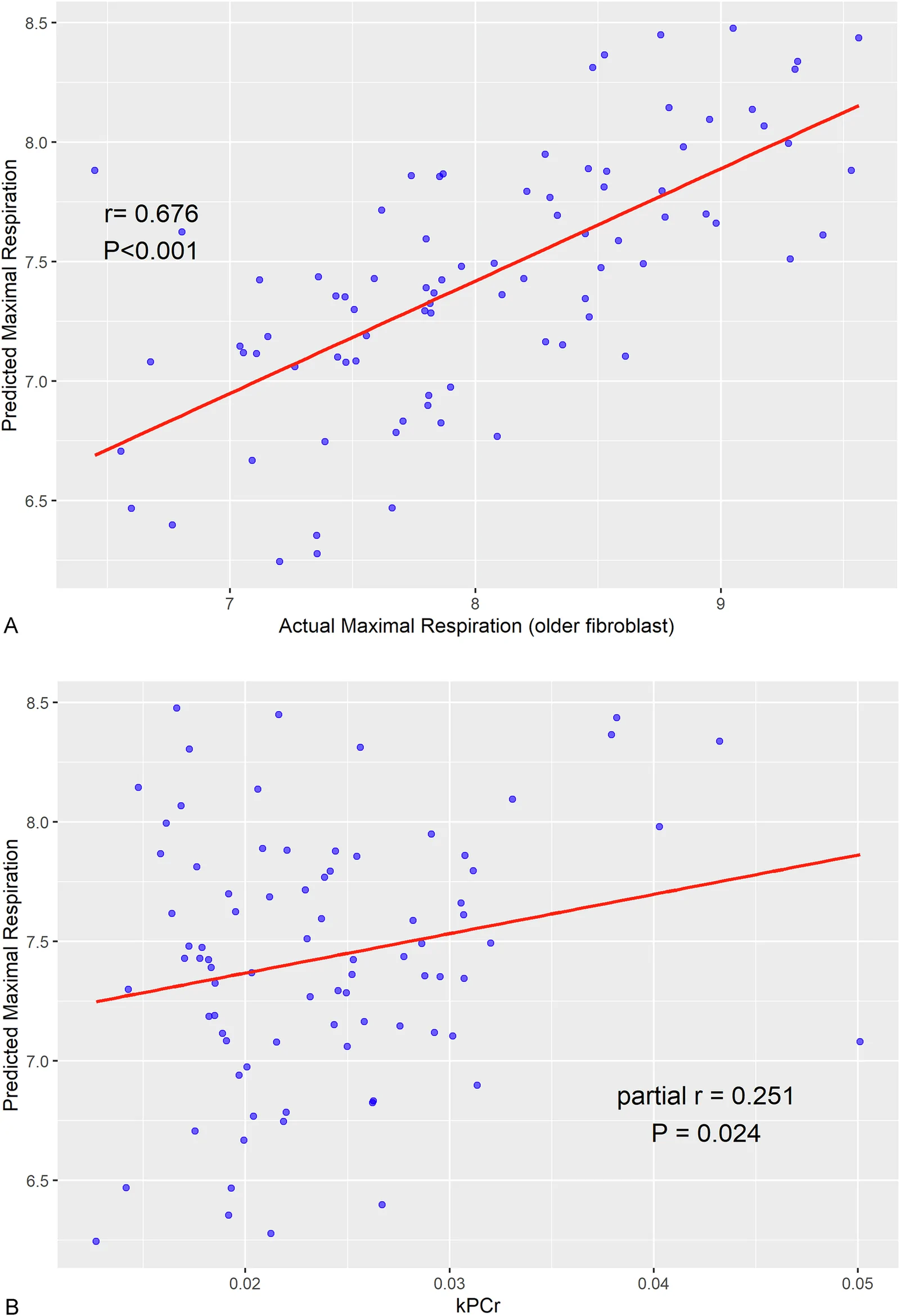
Validation of MitoAge. **A** Correlation between the actual (observed) maximal respiration from serum treatment in older fibroblasts compared to the predicted maximal respiration of the young fibroblast donor using the proteomic signature. **B** Correlation between the actual (observed) in vivo kPCr via ^31^P-MRS compared to the predicted maximal respiration of the young fibroblast donor using MitoAge.

We sought to validate MitoAge by using independent measures of bioenergetic capacity within the test sample. Therefore, we examined the association between MitoAge and the bioenergetic effect of the serum sample on fibroblasts derived from the older individual. We observed a positive correlation between the MitoAge and the actual observed respiration of the serum-treated fibroblasts from the older donor (R = 0.73, *P* < 0.001; Fig. 5A**;** Table 4).

**Table 4:** Associations of bioenergetic and clinical parameters with MitoAge

	MitoAge unadjusted		Bioenergetics (33 y.o. fibroblast donor)	
	*R*	*P*	*R*	*P*
Bioenergetics (72 y.o.)	0.676	<0.001	0.828	<.001
kPCr*	0.251	0.024	0.244	0.028
pVO_2_	0.241	0.045	0.221	0.066
Grip Strength	-0.008	0.944	–0.048	0.667

The correlations between MitoAge and (1) serum-mediated maximal respiration of the old fibroblast donor, (2) ^31^P-MRS, (3) pVO_2_, and (4) grip strength are described. These are compared with the associations with serum-mediated maximal respiration of the young fibroblast donor. *kPCR via ^31^P-MRS is partialed for PCr depletion.

### Prediction of in vivo mitochondrial function

We also used the measure of maximum oxidative capacity (quantified via ^31^P-MRS as post-exercise recovery rate of phosphocreatine “kPCr”; Fig. 5B) to determine if MitoAge was associated with an in vivo measure of mitochondrial function. Our results indicated that MitoAge was positively correlated to kPCr (R = 0.25, *P* = 0.003; Fig. 5B; Table 4).

### MitoAge is associated with cardiorespiratory fitness, but not strength

We evaluated correlations between MitoAge and measures of physical function, such as pVO_2_, and grip strength (Table 4). MitoAge had a positive correlation with pVO_2_ (R = 0.241, *P* = 0.045). We observed no correlation between MitoAge and grip strength (R = 0.022, *P* = 0.843).

## Discussion

The results of this study demonstrate some compounds in serum can actively mediate age-related related bioenergetic differences in cultured fibroblasts regardless of the intrinsic age of the target cell. The negative relationship between the age of the GESTALT serum donor and fibroblast OCR post-serum treatment was similar regardless of fibroblast donor age. This is in line with the premise that differences in mitochondrial bioenergetics are primarily driven by non-cellular factors present in circulation^15^. It is notable that correlations between the bioenergetic effects of serum are more strongly related to the participants’ biological age, based on epigenetic clocks, rather than the chronological of the serum donor. This is supported by our finding that chronological age and the Horvath clock had similar correlations to the bioenergetic effects of serum. We believe this may be the case as first generation of epigenetic clocks (such as Horvath and Hannum) were trained to predict chronological age^32,35^. Second-generation clocks (such as DNAmPhenoAge and GrimAge) were more strongly associated with the bioenergetic effects of serum regardless of tissue type. These second-generation clocks were designed to capture biological age by taking into account phenotypic differences and risks for mortality. DNAmPhenoAge was trained on markers of phenotypic age using clinical biomarkers, such as albumin, creatinine, and glucose^33^, while GrimAge was trained on various protein biomarkers and mortality risk factors, such as years of smoking history—all of which affect biological age^34,36^. These results add to a growing body of evidence that mitochondrial function may contribute to differences in biological aging.

It is also interesting to note the difference in strength of correlation between bioenergetics and epigenetic age among the tissues measured. The correlation between bioenergetics and the Horvath clock was similar among all tissues measured. This is likely due to the original, pan-tissue Horvath clock being trained on multiple tissues, therefore its age estimation is robust across multiple tissues. The other clocks were not necessarily designed to be pan-tissue, which is why there may be more subtle differences in age estimation across tissue types. In comparison, the correlation between bioenergetics and GrimAge was quite different among tissues. Of all correlations, GrimAge from muscle tissue had the strongest negative correlation with bioenergetics compared to that of PBMCs or monocytes. It is possible that this is the case because GrimAge includes protein surrogates, such as leptin and GDF-15, which may have greater age-related mitochondrial and metabolic relevance, especially in a more metabolically active tissues such as skeletal muscle.

Our univariate and multivariate analyses both identified proteins associated with the bioenergetic effects of the serum. Of the proteins that were common to both analyses, some have known functions in aging biology, mitochondrial function, and intercellular signaling, further supporting the idea that these circulating proteins can have broad, systemic effects on a wide range of tissues. AREG, for example, is a ligand of the EGF receptor and is associated with delayed cell death^37^ and senescence in certain cell types^38^. SMAD3 is an intracellular signal transducer and transcriptional modulator that was found to be reduced in older animals compared to young^39^. Age-related changes in SMAD3 signaling can impact ROS production and has implications in neurodegeneration^39^. Age-related SMAD3 signaling has also been associated with cell senescence^40^ and pro-inflammatory factors^41^.

While 11 proteins identified in this study were common to both univariate and multivariate analyses, many significant proteins were unique to each list. These differences arise from the methods used in each approach: the multivariate analysis takes into account the simultaneous effects of multiple independent variables, thereby affecting the impact of individual explanatory variables on the response. It is important to recognize that most proteins were identified by one method, but not both. For example, MCP-1 was the protein identified by the elastic net to have the strongest association with serum-mediated bioenergetics, and was not identified by the univariate approach. MCP-1 modulates innate immune response and inflammatory defense and has also been shown in animal models to decrease lifespan and promote metabolic dysregulation^42,43^. AKR7A2, identified by our univariate approach, has a strong association with bioenergetics, but was not identified by the elastic net. AKR7A2 also has metabolic effects associated with aging, such as in mediating oxidative stress^44,45^. Both MCP-1 and AKR7A2 are implicated in age-related metabolic changes; therefore, our comprehensive approach using both univariate and multivariate methods allows us to identify proteins that may be missed by using only one method.

Of the 25 proteins identified in our signature, only two were previously identified in Tanaka et al.’s plasma proteomic signature of age, CTSD and ectodysplasin-A (EDA), which utilized proteomic data from the same study^24^. It is reasonable that some proteins would be identified in both signatures. CTSD is associated with cognitive aging and neurodegeneration^46,47^ and EDA is associated with pro-inflammatory phenotypes and muscle wasting associated with aging^48,49^. The fact that only two out of the 25 proteins identified in our signature were previously identified in a chronological aging signature further corroborates the results of our mediation analysis showing that MitoAge mediates 91% of the effect of age on bioenergetics, with chronological age alone only accounting for 9% of this effect.

In this study, we demonstrate that circulating proteins can drive differences in mitochondrial function associated with age. This recognition enabled us to develop MitoAge as a powerful predictor of the bioenergetic effects of circulating factors that is also associated with complementary bioenergetic outcomes, including the recovery of ATP in skeletal muscle after exercise-induced depletion. Aligned with previous studies that have reported relationships between mitochondrial bioenergetic capacity with aging and physical performance^29,30,50^, results presented here demonstrate that MitoAge is similarly associated with pVO_2_. Interestingly, MitoAge was not associated with grip strength, suggesting that strength may be most closely related to other factors associated with advancing age. Overall, MitoAge and the findings presented here align with previous literature indicating the strong interdependent relationship between mitochondrial function and physical fitness^30,51,52^. Importantly, our analyses demonstrate that MitoAge can be utilized as a reliable biomarker of mitochondrial health in the context of human aging and functional decline.

This study is not without limitations. While this study utilizes cross-sectional serum samples from individuals of different ages, future studies may utilize longitudinal samples derived from individuals as they advance in age. Using MitoAge longitudinally could reflect the heterogeneity of aging trajectories both within and across individuals. Furthermore, larger studies applying MitoAge to larger cohorts are needed to investigate potential sex differences. Similar to other promising biomarkers of aging, the development of sex-specific markers and signatures should be evaluated.

One important advantage of the serum-exposure approach is its scalability for large population-based studies. Direct measurement of cellular bioenergetics across large cohorts may be impractical because each participant sample must be processed individually, increasing both logistical burden and susceptibility to batch variability. In contrast, serum-mediated bioenergetic assays allow measurements to be performed within a standardized experimental system and, when feasible, within a single batch, thereby reducing technical variability and improving reproducibility across large datasets. This feature is particularly relevant for implementation in large cohort studies such as GESTALT and may facilitate broader application in future epidemiologic and aging studies.

Among the major innovations presented here is the use of a biologically-informed and hypothesis-driven computational approach to develop MitoAge. This study introduces a new proteomic signature built upon a widely recognized cellular hallmark of aging: mitochondrial dysfunction. Reliable markers that can inform on cellular hallmarks of aging represent a new generation of aging biomarkers. There are many known contributors to alterations in age-related mitochondrial OXPHOS, such as diet, exercise, fitness, environmental exposures, and cognitive ability^16,18,29,30,52,53^. MitoAge can be used across multiple studies to inform on the role of age-related bioenergetic differences and broaden our understanding of drivers of health and longevity. Given the increasing prevalence of proteomic data from human cohort studies and clinical trials, MitoAge represents a scalable and cost effective way to support new analyses focused on mitochondrial health. Further, MitoAge can be used as an outcome in clinical trials of agents designed to promote healthy longevity by targeting mitochondrial dysfunction.

## Methods

### Study population

The GESTALT study began in 2015 with the goal of discovering novel biomarkers of human aging. The study enrolled healthy adults aged 20 years and older who were free of major and chronic diseases. Inclusion criteria and participant demographics have been previously described^24^. The GESTALT study was approved by the Institutional Review Board of the National Institutes of Health (IRB approval # 15-AG-0063). Informed consent at the time of each visit as well as the consent to publish the data collected was obtained from every participant. In addition, for the epigenetic clock data presented here, which is based on DNA methylation studies, all participants were required to consent to DNA/RNA testing and storage at all visits in order to participate in the study. The analysis was conducted in 88 participants with serum samples and plasma proteomic data.

### Cell culture and serum treatment

To examine the change in mitochondrial function mediated by serum, we treated two lines of primary human dermal fibroblasts from the San Diego Nathan Shock Center^54,55^ with 88 dilute serum samples from GESTALT participants. The two fibroblast lines were from male donors, aged 33 and 72 years old. Fibroblasts were cultured in high glucose DMEM (Thermo Scientific, 11965092), supplemented with 15% FBS, 1% NEAA, and 1% GlutaMAX. For serum treatment, the experimental design was adapted from Gonzalez‑Armenta et al.^16^. To summarize, cells were seeded in an Agilent Seahorse XFe96 assay plate at 20 K cells/well in 160 μL culture media. Following cell seeding, plates were allowed to rest for 45 min to 1 h at room temperature before incubating at 37 °C and 5% CO_2_ overnight. The next day, culture media was removed from each well, and 10% GESTALT serum (total in-well volume of 160 μL) in culture media was added to each well in quadruplicate. Fibroblasts with serum treatment were incubated for 24 h before bioenergetic assays. All experiments were conducted between in vitro passage 9-10, and no morphological differences were observed between passages. Cell number per well was quantified using a Gen5 brightfield imager (Agilent, Inc., Santa Clara, CA) pre- and post-treatment, and these quantifications were confirmed by Hoechst nuclear staining. The serum samples used for these exposure assays had corresponding plasma samples, which were used for proteomic analysis (described below).

### Respirometry

To examine the mitochondrial effects of serum treatments, we performed high-throughput respirometry (HTR) on primary human fibroblasts as previously described^16,17^. Respirometric analyses were conducted using an Agilent Seahorse XFe96 extracellular flux analyzer (XFe96; Agilent, Inc., Santa Clara, CA). The XF assay media contained XF DMEM Base Medium (Agilent Technologies, 103575-100) supplemented with 1 μM glucose (Agilent Technologies, 103577-100), 1 μM GlutaMAX (Thermo Scientific, 35050061), and 1 μM sodium pyruvate (Thermo Scientific, 11360070). The mitochondrial stress test Seahorse assay was conducted with final in-well concentrations of 1.25 μM oligomycin, 1 μM FCCP, 1.0 μM Antimycin A, 1.0 μM Rotenone, and 32.4 μM Hoechst (nuclear DNA stain; 30-min incubation). We included control treatments in each plate where cells were cultured either without serum or with 10% FBS. The Seahorse analyzer is calibrated before each assay and is maintained regularly.

### DNA methylation

Methylation data were gathered from multiple cell types from the GESTALT participants: peripheral blood mononuclear cells (PBMCs), monocytes, and skeletal muscle. The DNA methylation platform used for these analyses was Illumina EPICv1 (human). Blood collection and DNA methylation analysis was conducted as previously described^56^. Muscle biopsy preparation was also conducted as previously described^57^. Raw DNA methylation data files were processed, annotated, and analyzed using the *SeSAMe* Bioconductor package’s default settings^58,59^. Methylation data files were then submitted to the Clock Foundation’s DNA Methylation Age calculator (https://projects.clockfoundation.org/, accessed 2/27/2024), where key outputs were: Original (pan-tissue) Horvath Age^32^, DNAmPhenoAge^33^, and GrimAge^34^.

### Post-exercise phosphocreatine recovery rate (kPCr)

The maximum oxidative capacity of the vastus lateralis (VL) muscle was measured by in vivo phosphorus magnetic resonance spectroscopy (^31^P-MRS) and analyzed as previously described^60–62^. Briefly, skeletal muscle oxidative capacity was quantified via ^31^P-MRS as post-exercise recovery rate of phosphocreatine (kPCr), which was calculated as the inverse of the PCr exponential recovery time constant, τ (kPCr= 1/τPCr). These values were then adjusted for PCr depletion.

### Functional assessment

Cardiorespiratory fitness was assessed by maximum oxygen consumption during peak exercise (peak VO_2_ or pVO_2_) during a modified treadmill Balke test^44^. Gas exchange analyzer (MedGraphics, St. Paul, MN, USA) was used to capture the consumed O_2_ and expired CO_2_ concentrations, and oxygen consumption was calculated every 30 seconds. The highest oxygen consumption is the recorded peak VO_2_. Grip strength was measured using a hand-held dynamometer 3 times for each hand. The highest of each measure was used in the analysis.

### Proteomics

Plasma proteomic profiles were assessed using SomaScan 1.3 K as previously described^24,63–65^. In brief, this is a multiplexed, aptamer (SOMAmer)-based assay that simultaneously measures thousands of human proteins broadly ranging from femto- to micro-molar concentrations. This technology relies on protein-capture SOMAmer (Slow Off-rate Modified Aptamer) reagents^66^ designed to optimize high affinity, slow off-rate, and high specificity to target proteins. Proteins analyzed cover major molecular functions including receptors, kinases, growth factors, and hormones, and span a diverse collection of secreted, intracellular, and extracellular proteins or domains. The final data included relative abundances of 1305 SOMAmers or proteins expressed as relative fluorescence units (RFU). We normalized the raw protein abundance data by natural log-transformation, removing outliers outside 4 SD (0.47%), and imputing using k-nearest neighbor (KNN). These normalization steps account for hybridization, inter-sample, and inter-plate technical differences^24,64^.

### Data processing and univariate statistical analysis

Correlation coefficients between respiratory parameters and chronological age of GESTALT serum donor, epigenetic age of GESTALT serum donor, or other clinical measurements (Figs. 1–2, Table 4) were determined using Pearson’s correlation. The association between individual SOMAmer abundance and bioenergetic effects of serum were computed using multivariable regression models (SOMAmer abundance = independent variable; bioenergetic effect = dependent variable) adjusted for sex and SomaScan batch (Fig. 3, Table 1). A Bonferroni corrected *P* value of 3.83 × 10^−5^ (0.05/1305) was considered significant for the analysis of 1305 SOMAmers to adjust significance for multiple comparisons.

### Construction of proteomic signature of systemic mitochondrial health (MitoAge)

We constructed a proteomic predictor of serum-mediated bioenergetics by fitting elastic net regression models using the eNetXplorer R package, version 1.1.3, publicly available at https://github.com/juliancandia/eNetXplorer^67^. eNetXplorer evaluates elastic net model families across values of the mixing parameter α, ranging from ridge regression (α = 0) to lasso regression (α = 1). We evaluated α from 0 to 1 in increments of 0.1. The regularization parameter λ was selected within 500 repeated five-fold cross-validation runs by maximizing the out-of-bag quality function. Each model was compared with a null-model ensemble generated from 125 response-label permutations per cross-validation run. Participant sex was included as an unpenalized covariate. Protein features were summarized across repeated cross-validation iterations for the selected α model and compared with corresponding null-model distributions. Proteins with empirical feature-level *P* < 0.05, reflecting coefficient magnitude greater than expected under the null model, were retained as the MitoAge proteomic signature (Table 2).

Protein weights and predicted values were derived from a glmnet ridge regression. Using the selected proteins and weights, MitoAge was created as a linear combination of the products of the weight and the protein abundances. To evaluate the precision of MitoAge, we then examined the association between the predicted and actual serum-mediated bioenergetics. We further evaluated the association of MitoAge with serum-mediated bioenergetics in a separate fibroblast line as well as with an in vivo measures of mitochondrial OXPHOS (^31^P-MRS) with Pearson’s correlation. To evaluate how well MitoAge reflects additional age-related functional measures, we examined the association of MitoAge with pVO_2_ and grip strength, both functional measures known to decline with age. Our goal with MitoAge was to develop a signature of age-related mitochondrial health, participant chronological age was not included as a covariate. To address how age may affect the model, we performed a causal mediation analysis to examine whether MitoAge mediated the association between age and bioenergetics. The mediator model regressed the MitoAge on age, and the outcome model regressed bioenergetics on both age and MitoAge. Indirect, direct, and total effects were estimated using nonparametric bootstrapping with 5,000 simulations and percentile-based 95% confidence intervals. Analyses were performed in R using the mediation package.

## Supplementary information

Heimler et al_Supplemental Figures and Tables

Supplementary Data 1

## Data Availability

Data is provided within the manuscript or supplementary information files. Additional data files may be available upon request.

## References

[1] López-Otín, C., Blasco, M. A., Partridge, L., Serrano, M. & Kroemer, G. Hallmarks of aging: An expanding universe. *Cell* **186**, 243–278 (2023). 10.1016/j.cell.2022.11.001 36599349

[2] Serviddio, G. et al. Bioenergetics in aging: mitochondrial proton leak in aging rat liver, kidney and heart. *Redox Rep.* **12**, 91–95 (2007). 10.1179/135100007X162112 17263918

[3] Kujoth, G. C. et al. Mitochondrial DNA mutations, oxidative stress, and apoptosis in mammalian Aging. *Science* **309**, 481–484 (2005). 10.1126/science.1112125 16020738

[4] Carey, B. W., Finley, L. W. S., Cross, J. R., Allis, C. D. & Thompson, C. B. Intracellular α-ketoglutarate maintains the pluripotency of embryonic stem cells. *Nature* **518**, 413–416 (2015). 10.1038/nature13981 PMC4336218 25487152

[5] Kaplon, J. et al. A key role for mitochondrial gatekeeper pyruvate dehydrogenase in oncogene-induced senescence. *Nature* **498**, 109–112 (2013). 10.1038/nature12154 23685455

[6] Tian, Q. et al. Muscle mitochondrial energetics predicts mobility decline in well-functioning older adults: The baltimore longitudinal study of aging. *Aging Cell* **21**, e13552 (2022). 10.1111/acel.13552 PMC8844110 35048491

[7] Tian, Q. et al. Skeletal muscle mitochondrial function predicts cognitive impairment and is associated with biomarkers of Alzheimer’s disease and neurodegeneration. *Alzheimers Dement J. Alzheimers Assoc.* **19**, 4436–4445 (2023). 10.1002/alz.13388 PMC10592411 37530130

[8] Tyrrell, D. J., Bharadwaj, M. S., Jorgensen, M. J., Register, T. C. & Molina, A. J. A. Blood cell respirometry is associated with skeletal and cardiac muscle bioenergetics: Implications for a minimally invasive biomarker of mitochondrial health. *Redox Biol.* **10**, 65–77 (2016). 10.1016/j.redox.2016.09.009 PMC5045569 27693859

[9] Tyrrell, D. J. et al. Blood-based bioenergetic profiling reflects differences in brain bioenergetics and metabolism. *Oxid. Med Cell Longev.* **2017**, 7317251 (2017). 10.1155/2017/7317251 PMC5643153 29098063

[10] Braganza, A. et al. Platelet bioenergetics correlate with muscle energetics and are altered in older adults. *JCI Insight* **4**, e128248 (2019). 10.1172/jci.insight.128248 PMC6629251 31120438

[11] Brack, A. S. et al. Increased Wnt signaling during aging alters muscle stem cell fate and increases fibrosis. *Science* **317**, 807–810 (2007). 10.1126/science.1144090 17690295

[12] Conboy, I. M. et al. Rejuvenation of aged progenitor cells by exposure to a young systemic environment. *Nature* **433**, 760–764 (2005). 10.1038/nature03260 15716955

[13] Loffredo, F. S. et al. Growth differentiation factor 11 is a circulating factor that reverses age-related cardiac hypertrophy. *Cell* **153**, 828–839 (2013). 10.1016/j.cell.2013.04.015 PMC3677132 23663781

[14] Salpeter, S. J. et al. Systemic regulation of the age-related decline of pancreatic β-cell replication. *Diabetes* **62**, 2843–2848 (2013). 10.2337/db13-0160 PMC3717843 23630298

[15] Gonzalez-Armenta, J. L., Li, N., Lee, R. L., Lu, B. & Molina, A. J. A. Heterochronic parabiosis: Old blood induces changes in mitochondrial structure and function of young mice. *J. Gerontol. A Biol. Sci. Med Sci.* **76**, 434–439 (2021). 10.1093/gerona/glaa299 PMC8177798 33377482

[16] Gonzalez-Armenta, J. L. et al. Serum factors mediate changes in mitochondrial bioenergetics associated with diet and exercise interventions. *GeroScience* **46**, 349–365 (2023). 10.1007/s11357-023-00855-w PMC10828137 37368157

[17] Amick, K. A. et al. Plasma glycocholic acid and linoleic acid identified as potential mediators of mitochondrial bioenergetics in Alzheimer’s dementia. *Front Aging Neurosci.* **14**, 954090 (2022). 10.3389/fnagi.2022.954090 PMC9540364 36212044

[18] Heimler, S. R. et al. Nervonic acid and 15-epi-PGA1 mediate systemic mitochondrial dysfunction in AD dementia. *GeroScience*. 10.1007/s11357-025-01776-6 (2025). PMC12972455 40640460

[19] Kawanishi, N. et al. Effects of aging on serum levels of lipid molecular species as determined by lipidomics analysis in Japanese men and women. *Lipids Health Dis.* **17**, 135 (2018). 10.1186/s12944-018-0785-6 PMC5991438 29875018

[20] Montoliu, I. et al. Serum profiling of healthy aging identifies phospho- and sphingolipid species as markers of human longevity. *Aging* **6**, 9–25 (2014). 10.18632/aging.100630 PMC3927806 24457528

[21] Yu, Z. et al. Human serum metabolic profiles are age dependent. *Aging Cell* **11**, 960–967 (2012). 10.1111/j.1474-9726.2012.00865.x PMC3533791 22834969

[22] Menni, C. et al. Circulating proteomic signatures of chronological age. *J. Gerontol. A Biol. Sci. Med Sci.* **70**, 809–816 (2015). 10.1093/gerona/glu121 PMC4469006 25123647

[23] Sebastiani, P. et al. Protein signatures of centenarians and their offspring suggest centenarians age slower than other humans [Internet]. 10.1111/acel.13290. PMC7884029 33512769

[24] Tanaka, T. et al. Plasma proteomic signature of age in healthy humans. *Aging Cell* **17**, e12799 (2018). 10.1111/acel.12799 PMC6156492 29992704

[25] Sun, B. B. et al. Genomic atlas of the human plasma proteome. *Nature* **558**, 73–79 (2018). 10.1038/s41586-018-0175-2 PMC6697541 29875488

[26] Tanaka, T. et al. Plasma proteomic biomarker signature of age predicts health and life span. *eLife* **9**, e61073 (2020). 10.7554/eLife.61073 PMC7723412 33210602

[27] Baird, A. L., Westwood, S. & Lovestone, S. Blood-based proteomic biomarkers of Alzheimer’s disease pathology. *Front Neurol.* **6**, 236 (2015). 10.3389/fneur.2015.00236 PMC4644785 26635716

[28] Di Narzo, A. F. et al. High-throughput characterization of blood serum proteomics of IBD patients with respect to aging and genetic factors. *PLoS Genet* **13**, e1006565 (2017). 10.1371/journal.pgen.1006565 PMC5271178 28129359

[29] Tyrrell, D. J. et al. Blood-cell bioenergetics are associated with physical function and inflammation in overweight/obese older adults. *Exp. Gerontol.* **70**, 84–91 (2015). 10.1016/j.exger.2015.07.015 PMC6471587 26226578

[30] Tyrrell, D. J. et al. Respirometric profiling of muscle mitochondria and blood cells are associated with differences in gait speed among community-dwelling older adults. *J. Gerontol. A Biol. Sci. Med Sci.* **70**, 1394–1399 (2015). 10.1093/gerona/glu096 PMC4731403 25030980

[31] Kramer, P. A. et al. Skeletal muscle energetics explain the sex disparity in mobility impairment in the study of muscle, mobility and aging. *J. Gerontol. Ser. A* **79**, glad283 (2024). 10.1093/gerona/glad283 PMC10960628 38150179

[32] Horvath, S. DNA methylation age of human tissues and cell types. *Genome Biol.* **14**, R115 (2013). 10.1186/gb-2013-14-10-r115 PMC4015143 24138928

[33] Levine, M. E. et al. An epigenetic biomarker of aging for lifespan and healthspan. *Aging* **10**, 573–591 (2018). 10.18632/aging.101414 PMC5940111 29676998

[34] Lu, A. T. et al. DNA methylation GrimAge strongly predicts lifespan and healthspan. *Aging* **11**, 303–327 (2019). 10.18632/aging.101684 PMC6366976 30669119

[35] Hannum, G. et al. Genome-wide methylation profiles reveal quantitative views of human aging rates. *Mol. Cell* **49**, 359–367 (2013). 10.1016/j.molcel.2012.10.016 PMC3780611 23177740

[36] McCrory, C. et al. GrimAge outperforms other epigenetic clocks in the prediction of age-related clinical phenotypes and all-cause mortality. *J. Gerontol. Ser. A* **76**, 741–749 (2021). 10.1093/gerona/glaa286 PMC8087266 33211845

[37] Xu, Q. et al. Targeting amphiregulin (AREG) derived from senescent stromal cells diminishes cancer resistance and averts programmed cell death 1 ligand (PD-L1)-mediated immunosuppression. *Aging Cell* **18**, e13027 (2019). 10.1111/acel.13027 PMC6826133 31493351

[38] Pommer, M., Kuphal, S. & Bosserhoff, A. K. Amphiregulin Regulates Melanocytic Senescence. *Cells* **10**, 2 (2021). 10.3390/cells10020326 PMC7914549 33562468

[39] Tichauer, J. E. et al. Age-dependent changes on TGFβ1 Smad3 pathway modify the pattern of microglial cell activation. *Brain Behav. Immun.* **37**, 187–196 (2014). 10.1016/j.bbi.2013.12.018 PMC3951654 24380849

[40] Gao, X. Y. et al. Acetyltransferase p300 regulates atrial fibroblast senescence and age-related atrial fibrosis through p53/Smad3 axis. *Aging Cell* **22**, e13743 (2023). 10.1111/acel.13743 PMC9835568 36468256

[41] Liu, Y., Yu, L., Xu, Y., Tang, X. & Wang, X. Substantia nigra Smad3 signaling deficiency: relevance to aging and Parkinson’s disease and roles of microglia, proinflammatory factors, and MAPK. *J. Neuroinflamm.* **17**, 342 (2020). 10.1186/s12974-020-02023-9 PMC7670688 33198771

[42] Luciano-Mateo, F. et al. Systemic overexpression of C-C motif chemokine ligand 2 promotes metabolic dysregulation and premature death in mice with accelerated aging. *Aging* **12**, 20001–20023 (2020). 10.18632/aging.104154 PMC7655213 33104522

[43] Guo, D., Zhu, W. & Qiu, H. C-C motif chemokine ligand 2 and chemokine receptor 2 in cardiovascular and neural aging and aging-related diseases. *Int J. Mol. Sci.* **25**, 8794 (2024). 10.3390/ijms25168794 PMC11355023 39201480

[44] Ahmed, M. M. E. et al. Aldo-keto reductase-7A protects liver cells and tissues from acetaminophen-induced oxidative stress and hepatotoxicity. *Hepatology* **54**, 1322–1332 (2011). 10.1002/hep.24493 21688283

[45] Wang, Q. et al. Glycyrrhizic acid ameliorates hepatic fibrosis by inhibiting oxidative stress via AKR7A2. *Phytomedicine* **133**, 155878 (2024). 10.1016/j.phymed.2024.155878 39121535

[46] Vidoni, C., Follo, C., Savino, M., Melone, M. A. B. & Isidoro, C. The role of cathepsin D in the pathogenesis of human neurodegenerative disorders. *Med Res Rev.* **36**, 845–870 (2016). 10.1002/med.21394 27114232

[47] Urbanelli, L. et al. Cathepsin D expression is decreased in Alzheimer’s disease fibroblasts. *Neurobiol. Aging* **29**, 12–22 (2008). 10.1016/j.neurobiolaging.2006.09.005 17049675

[48] Barbera, M. C. et al. Increased ectodysplasin-A2-receptor EDA2R is a ubiquitous hallmark of aging and mediates parainflammatory responses. *Nat. Commun.* **16**, 1898 (2025). 10.1038/s41467-025-56918-3 PMC11847917 39988718

[49] Özen, S. D. & Kir, S. Ectodysplasin A2 receptor signaling in skeletal muscle pathophysiology. *Trends Mol. Med.* **30**, 471–483 (2024). 10.1016/j.molmed.2024.02.002 38443222

[50] Heimler, S. et al. Platelet bioenergetics are associated with resting metabolic rate and exercise capacity in older adult women [Internet]. 10.26124/BEC:2022-0002 (2022).

[51] Effects of physical activity and age on mitochondrial function | QJM: An International Journal of Medicine | Oxford Academic [Internet]. Available from: https://academic.oup.com/qjmed/article-abstract/89/4/251/1499967 [cited 2025 Apr 30].

[52] Grevendonk, L. et al. Impact of aging and exercise on skeletal muscle mitochondrial capacity, energy metabolism, and physical function. *Nat. Commun.* **12**, 4773 (2021). 10.1038/s41467-021-24956-2 PMC8346468 34362885

[53] Reddam, A., McLarnan, S. & Kupsco, A. Environmental chemical exposures and mitochondrial dysfunction: A review of recent literature. *Curr. Environ. Health Rep.* **9**, 631–649 (2022). 10.1007/s40572-022-00371-7 PMC9729331 35902457

[54] Shadel, G. S. et al. The San Diego Nathan Shock Center: Tackling the heterogeneity of aging. *GeroScience* **43**, 2139–2148 (2021). 10.1007/s11357-021-00426-x PMC8599742 34370163

[55] Phang, H. J. et al. Protocol for the San Diego Nathan Shock Center Clinical Cohort: A new resource for studies of human aging. *BMJ Open* **14**, e082659 (2024). 10.1136/bmjopen-2023-082659 PMC11202663 38925692

[56] Roy, R. et al. Epigenetic signature of human immune aging in the GESTALT study. *eLife* **12**, e86136 (2023). 10.7554/eLife.86136 PMC10506794 37589453

[57] Ubaida-Mohien, C. et al. Discovery proteomics in aging human skeletal muscle finds change in spliceosome, immunity, proteostasis and mitochondria. *eLife* **8**, e49874 (2019). Kaeberlein M, Rosen CJ, Kaeberlein M, Anderson R, editors. 10.7554/eLife.49874 PMC6810669 31642809

[58] Zhou, W., Triche, T. J. Jr, Laird, P. W. & Shen, H. SeSAMe: reducing artifactual detection of DNA methylation by Infinium BeadChips in genomic deletions. *Nucleic Acids Res* **46**, e123 (2018). 10.1093/nar/gky691 PMC6237738 30085201

[59] Zhou, W., Laird, P. W. & Shen, H. Comprehensive characterization, annotation and innovative use of Infinium DNA methylation BeadChip probes. *Nucleic Acids Res* **45**, e22 (2017). 10.1093/nar/gkw967 PMC5389466 27924034

[60] Choi, S. et al. 31P Magnetic resonance spectroscopy assessment of muscle bioenergetics as a predictor of gait speed in the baltimore longitudinal study of aging [Internet]. Available from: 10.1093/gerona/glw059. PMC5106855 27075894

[61] Zane, A. C. et al. Muscle strength mediates the relationship between mitochondrial energetics and walking performance. *Aging Cell* **16**, 461–468 (2017). 10.1111/acel.12568 PMC5418194 28181388

[62] Naressi, A., Couturier, C., Castang, I., de Beer, R. & Graveron-Demilly, D. Java-based graphical user interface for MRUI, a software package for quantitation of in vivo/medical magnetic resonance spectroscopy signals. *Comput Biol. Med*. **31**, 269–286 (2001). 10.1016/s0010-4825(01)00006-3 11334636

[63] Gold, L. et al. Aptamer-based multiplexed proteomic technology for biomarker discovery. *PLOS ONE* **5**, e15004 (2010). 10.1371/journal.pone.0015004 PMC3000457 21165148

[64] Candia, J., Daya, G. N., Tanaka, T., Ferrucci, L. & Walker, K. A. Assessment of variability in the plasma 7k SomaScan proteomics assay. *Sci. Rep.* **12**, 17147 (2022). 10.1038/s41598-022-22116-0 PMC9561184 36229504

[65] Candia, J. et al. Assessment of variability in the SOMAscan assay. *Sci. Rep.* **7**, 14248 (2017). 10.1038/s41598-017-14755-5 PMC5660188 29079756

[66] Rohloff, J. C. et al. Nucleic acid ligands with protein-like side chains: Modified aptamers and their use as diagnostic and therapeutic agents. *Mol. Ther. Nucleic Acids* **3**, e201 (2014). 10.1038/mtna.2014.49 PMC4217074 25291143

[67] Candia, J. & Tsang, J. S. eNetXplorer: An R package for the quantitative exploration of elastic net families for generalized linear models. *BMC Bioinforma.* **20**, 189 (2019). 10.1186/s12859-019-2778-5 PMC6469092 30991955

