# Supplementary material for: The blood proteome predicts the impact of circulating factors on age-related mitochondrial health
Source: NPJ Aging. 2026 Jul 24;12(1):158. doi: 10.1038/s41514-026-00449-8 (PMC13759516; doi:10.1038/s41514-026-00449-8)
Supplement: Supplementary file 1 [file 41514_2026_449_MOESM1_ESM.pdf]

SUPPLEMENTARY FIGURES AND TABLES

Heimler et al. The blood proteome predicts the impact of circulating factors on age-related mitochondrial health. *NPJ Aging*, 2026.

Supplementary Figure 1: Maximal respiration, but not basal respiration, negatively correlates with the chronological age of the serum donor. N=88 GESTALT serum samples treated on human primary fibroblasts (fibroblast donor = male, 72 y.o).

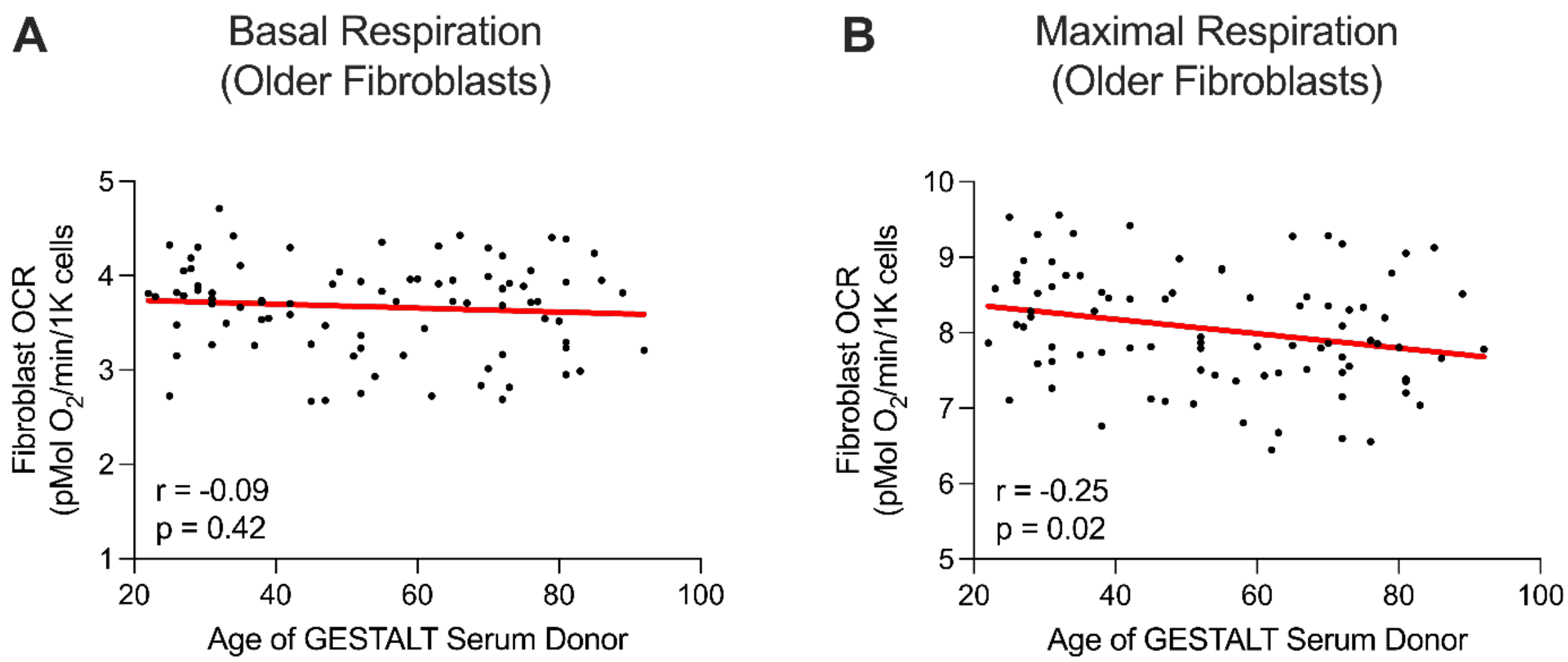

Supplementary Table 1: Partial correlations with chronological age.

	**Unadjusted R**	**Unadjusted P-value**	**Adjusted R**	**Adjusted P-value**
**PBMC_Horvath**	-0.242	0.025	0.308	0.019
**PBMC_Pheno**	-0.293	0.006	0.01	0.94
**PBMC_Grim**	-0.281	0.009	0.03	0.825
**Mono_Horvath**	-0.241	0.028	0.125	0.351
**Mono_Pheno**	-0.263	0.016	0.018	0.895
**Mono_Grim**	-0.266	0.015	-0.012	0.93
**Musc_Horvath**	-0.24	0.056	0.095	0.478
**Musc_Pheno**	-0.273	0.029	-0.035	0.792
**Musc_Grim**	-0.374	0.002	-0.179	0.179
